# Trends in postpartum hemorrhage in high resource countries: a review and recommendations from the International Postpartum Hemorrhage Collaborative Group

**DOI:** 10.1186/1471-2393-9-55

**Published:** 2009-11-27

**Authors:** Marian Knight, William M Callaghan, Cynthia Berg, Sophie Alexander, Marie-Helene Bouvier-Colle, Jane B Ford, KS Joseph, Gwyneth Lewis, Robert M Liston, Christine L Roberts, Jeremy Oats, James Walker

**Affiliations:** 1 National Perinatal Epidemiology Unit, University of Oxford, Oxford, UK; 2 Division of Reproductive Health, Centers for Disease Control and Prevention (CDC), Atlanta, GA, USA; 3 Perinatal Epidemiology and Reproductive Health Unit, Université Libre de Bruxelles, Belgium; 4 INSERM- Unité 149, Paris, France; 5 The Kolling Institute of Medical Research, University of Sydney at Royal North Shore Hospital, Sydney, Australia; 6 Dept of Obstetrics & Gynecology and Pediatrics, Dalhousie University, Halifax, Canada; 7 Department of Health, London, UK; 8 BC Women's Hospital & Health Centre, Vancouver, BC, Canada; 9 The Royal Women's Hospital, Parkville, Victoria, Australia; 10 St James University Hospital, Leeds, UK; 11 Dept of Obstetrics & Gynecology and the School of Population and Public Health, University of British Columbia, Vancouver, Canada

## Abstract

**Background:**

Postpartum hemorrhage (PPH) is a major cause of maternal mortality and morbidity worldwide. Several recent publications have noted an increasing trend in incidence over time. The international PPH collaboration was convened to explore the observed trends and to set out actions to address the factors identified.

**Methods:**

We reviewed available data sources on the incidence of PPH over time in Australia, Belgium, Canada, France, the United Kingdom and the USA. Where information was available, the incidence of PPH was stratified by cause.

**Results:**

We observed an increasing trend in PPH, using heterogeneous definitions, in Australia, Canada, the UK and the USA. The observed increase in PPH in Australia, Canada and the USA was limited solely to immediate/atonic PPH. We noted increasing rates of severe adverse outcomes due to hemorrhage in Australia, Canada, the UK and the USA.

**Conclusion:**

Key Recommendations

1. Future revisions of the International Classification of Diseases should include separate codes for atonic PPH and PPH immediately following childbirth that is due to other causes. Also, additional codes are required for placenta accreta/percreta/increta.

2. Definitions of PPH should be unified; further research is required to investigate how definitions are applied in practice to the coding of data.

3. Additional improvement in the collection of data concerning PPH is required, specifically including a measure of severity.

4. Further research is required to determine whether an increased rate of reported PPH is also observed in other countries, and to further investigate potential risk factors including increased duration of labor, obesity and changes in second and third stage management practice.

5. Training should be provided to all staff involved in maternity care concerning assessment of blood loss and the monitoring of women after childbirth. This is key to reducing the severity of PPH and preventing any adverse outcomes.

6. Clinicians should be more vigilant given the possibility that the frequency and severity of PPH has in fact increased. This applies particularly to small hospitals with relatively few deliveries where management protocols may not be defined adequately and drugs or equipment may not be on hand to deal with unexpected severe PPH.

## Background

Death as a consequence of pregnancy remains an important cause of premature mortality worldwide. An estimated 500,000 women die from this potentially preventable cause each year [1], with up to an estimated quarter of these deaths occurring as a consequence of hemorrhage [2]. Although the majority of these deaths occur in low income countries, several recent publications [3-6] have noted an increasing trend in the incidence of postpartum hemorrhage (PPH) over time in high resource countries. However, these studies used different data sources, differing definitions of postpartum hemorrhage and varying methodologies such that it is not clear if these results are widely generalisable and hence whether a similar pattern is likely to be observed in other high resource countries. The reasons for the observed increase also remain obscure. Confirmation of any trend, together with an investigation of potential associated factors is important in order to develop recommendations to address PPH in the future.

The aims of this study, conducted by the International PPH Collaborative Group, which included representatives from Australia, Belgium, Canada, France, the United Kingdom and the USA, were to investigate further whether such trends are observed in other countries with well developed health systems, to explore potential causes for any observed trends and to set out recommendations to improve available data on PPH as well as actions to address the factors identified.

## Methods

We reviewed available data sources on the incidence of PPH over time in Australia, Belgium, Canada, France, the United Kingdom and the USA. Where information was available, the incidence of PPH was stratified by cause (Table 1). Because of variations in definitions of PPH used between countries and between data sources, we did not restrict our analysis to PPH defined using any one particular classification. The definitions used in each country are set out below.

**Table 1:** Classifications and coding for causes of PPH

Cause of PPH	ICD-9 coding	ICD-10 Coding	Scottish data classification
Third stage hemorrhage (Postpartum hemorrhage due to retained placenta)	666.0	O72.0	Hemorrhage due to retained products

Other immediate postpartum hemorrhage, within the first 24 hours following delivery of placenta (Uterine atony)	666.1	O72.1	Hemorrhage due to uterine atony

Delayed and secondary postpartum hemorrhage (after the first 24 hours following delivery)	666.2	O72.2	

Postpartum coagulation defects	666.3	O72.3	

Other causes			Hemorrhage due to placental abruption, placenta previa, trauma or other causes

### Data sources

#### Australia

In New South Wales (NSW) data were obtained from the Admitted Patient Data Collection (APDC) a census of all hospital discharges from public and private hospitals [7]. Diagnoses and procedures obtained from the medical records for each hospitalization are coded according to the International Statistical Classification of Diseases and Related Health Problems, Australian Modification (ICD9 to July 1998 and ICD10 subsequently) and the affiliated Australian Classification of Health Interventions. Childbirth hospitalizations were identified by linkage of the hospital separation data with birth data.

Postpartum hemorrhage (PPH) cases were identified using the ICD codes outlined in Table 1. In the Australian Modification of the ICD, PPH is defined as a hemorrhage of 500 ml or more following vaginal delivery or 750 ml or more following a caesarean delivery resulting in a recorded clinical diagnosis of PPH and identified during the birth hospitalization from the hospital data. In NSW PPH in the hospital data is reported with 74% sensitivity and 99% specificity when compared with the medical record [8].

Data for Victoria was derived from the perinatal data collection form which is completed by the birth attendant (usually the midwife) for each birth. The form includes a check-box for PPH. The ICD-10AM definition of PPH was also used. The most recent validation of the accuracy of the data was carried out for births in 2003. Data relating to a random sample of 1% was collected from the original medical record and compared with that in the database of the Perinatal Data Collection Unit. PPH as determined from the estimated blood loss recorded in the medical record was coded as 'Yes' or 'No' and compared with the record of PPH in the perinatal data collection. Sensitivity was 83.6%; specificity 97.5%, PPV 76.7%, NPV 98.4% and accuracy 96.3% (unpublished data).

#### Belgium

Hospital discharge data for the Flanders region was examined to determine the proportion of women receiving a blood transfusion within 24 hr of birth as a proxy for PPH.

#### Canada

As described elsewhere [5], information for Canadian trends was based on all hospital deliveries as documented in the Discharge Abstract Database of the Canadian Institute for Health Information from 1991 to 2005. All medical diagnoses were coded using the International Classification of Diseases (ICD-9 up to 2000, and increasingly in ICD-10 from 2001 onwards), while procedures were coded using the Canadian Classification of Diagnostic, Therapeutic and Surgical Procedures (CCP) and the Canadian Classification of Interventions (CCI). PPH was coded if blood loss after childbirth exceeded 500 ml after a vaginal delivery and 1,000 ml after a caesarean delivery or if the physician made a notation of PPH in the medical chart. Data from Quebec, Manitoba and Nova Scotia were excluded because complete data for these provinces were not available for the entire study period.

Cases of postpartum hemorrhage were identified using ICD-9 codes 666.0, 666.1, 666.2 and 666.3 and ICD-10 codes O720, O721, O722 and O723 for postpartum hemorrhage due to retained placenta (third stage hemorrhage), uterine atony (immediate postpartum hemorrhage, within the first 24 hours following delivery of placenta), delayed and secondary postpartum hemorrhage (after the first 24 hours following delivery) and postpartum hemorrhage due to coagulation defects, respectively.

Women whose deliveries were complicated by postpartum hemorrhage and who additionally had an abdominal hysterectomy were identified using the relevant CCP and CCI codes. Blood transfusions were identified using a specific code introduced in the database in 1994 (blood transfusion rates were thus unavailable for 1991 to 1993).

A validation study using 2002 data (with information from a clinically focused database in the Canadian province of Nova Scotia as the gold standard) showed high rates of accuracy for the information on PPH in the Discharge Abstract Database [9]. The sensitivity and specificity rates for PPH were 90.2% and 98.2%, respectively, while the same rates for blood transfusion were 85.7% and 99.8%, respectively.

#### France

Published maternal mortality data were reviewed [10].

#### United Kingdom

Scottish data were derived from the Scottish Confidential Audit of Severe Maternal Morbidity [11]. A standardized, objective case assessment was developed to allow hospital clinical risk management teams to assess their own local cases of major obstetric hemorrhage. All consultant-led maternity units in Scotland participated in data collection.

UK data on postpartum hemorrhage, including data from England, Scotland, Wales and Northern Ireland, were obtained from the UK Obstetric Surveillance System (UKOSS) survey of hemorrhage-associated peripartum hysterectomy [12].

#### United States

Data for the United States were obtained from the Nationwide Inpatient Sample (NIS) for 1994-2006. The NIS is part of the Agency for Healthcare Research and Quality's Healthcare Cost and Utilization Project. The NIS [13] with appropriate weighting generates a nationally representative sample of inpatient hospital admissions. The database contains up to 15 diagnosis fields and 15 procedure fields; diagnoses and procedures are coded at the hospital at discharge using the ICD-9-CM. Except for age, the NIS does not collect individual demographic information nor does it report obstetrical characteristics for individual pregnancies except those that can be translated to ICD-9-CM codes. Hence, this report focused on diagnosis codes for postpartum hemorrhage and ICD-9-CM coded characteristics of the delivery. Delivery hospitalizations were identified using a previously published algorithm based on ICD-9-CM diagnosis and procedure codes and diagnosis-related group (DRG) codes [14].

### Ethics Committee Approval

The UK peripartum hysterectomy study was approved by the London Multi-centre Research Ethics Committee (ref 04/MRE02/73) and the study of New South Wales data was approved by the University of Sydney Human Research Ethics Committee (ref #7523). No other permissions were required for use of the data presented.

## Results

### Incidence

#### ICD-coded data

**Figure 1:**
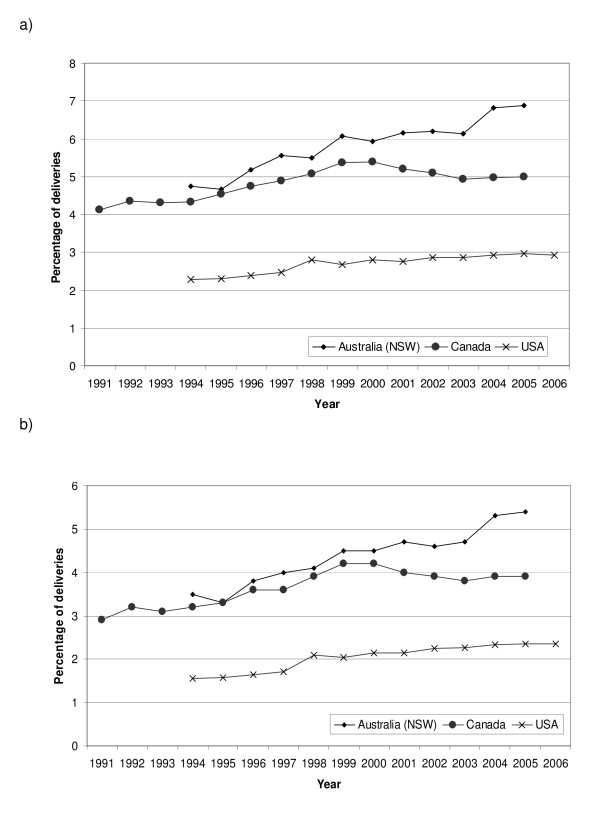
**Trends in a) all PPH and b) Atonic PPH obtained from coded data sources**.

We observed an increasing trend in coded PPH between 1991 and 2006 in Canada, New South Wales and the USA (figure 1a). The observed increase in coded PPH was limited solely to immediate/atonic PPH (figure 1b).

#### Other data

**Figure 2:**
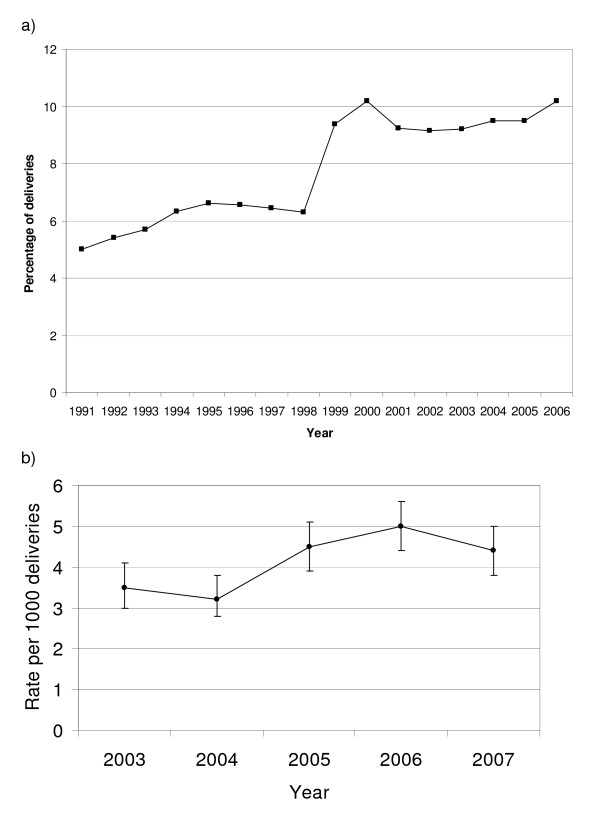
**Trends in PPH using other data a) PPH in Victoria, Australia and b) Severe obstetric hemorrhage in Scotland**.

An increase was also observed in PPH in Victoria between 1991 and 2006 (Figure 2a; note the stepped change between 1998/1999 is related to a change in definition of PPH from 600 mls to 500 mls) and severe obstetric hemorrhage in Scotland between 2003 and 2007, (figure 2b).

#### Severity

Increasing transfusion rates at childbirth were noted in the USA and Australia, but not in Canada or Flanders (Table 2) [5,15,16]. There was no significant increase in the rate of hysterectomy for peripartum hemorrhage between 1997-8 and 2005-6 in the UK [12,17], although in Canada rates of hysterectomy for atonic PPH increased from 24.0 per 100,000 deliveries in 1991 to 41.7 per 100,000 deliveries in 2004 (73% increase, 95% CI 27-137) [5]. Maternal mortality from hemorrhage appeared to be static (Australia between 1994-6 and 2003-5 [18,19], France between 1997-9 and 2000-2[10], UK between 1985-7 and 2003-5 [20], US between 1998-2004 [21]. Notably, Sheehan syndrome increased in Canada from 3.7 in 1991-93 to 12.6 per million deliveries in 2002-04 (241% increase, 95% CI 8% decrease to 1,158% increase p = 0.10; p value for increasing annual linear trend = 0.008) [5]; data were not available for other countries. There was no increase in maternal mortality from PPH and in blood transfusion for PPH in Canada.

**Table 2:** Blood transfusion within 24 hr of birth in Flanders, Belgium

Year	Number (%) of women transfused	Number of births
2002	715 (1.2)	60043

2003	679 (1.1)	60406

2004	719 (1.1)	62654

2005	670 (1.0)	64222

2006	763 (1.2)	65933

2007	814 (1.2)	67037

#### Associated factors

**Figure 3:**
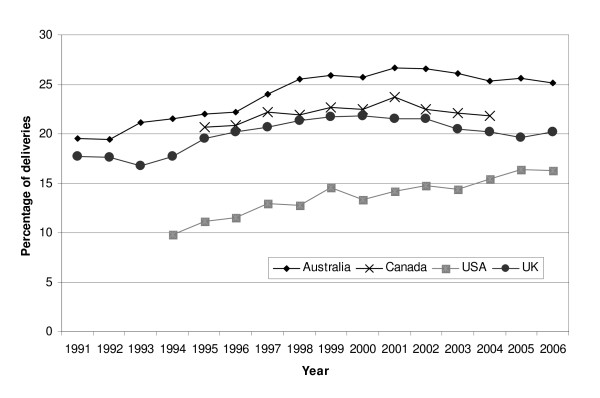
**Proportion of deliveries with induced labor**. Note that the sources of data and definitions of induction used differ between countries and these data are included to show temporal trends and not for the purposes of inter-country comparison of rates.

In all populations examined, maternal age at childbirth was increasing [22-25], caesarean delivery was becoming more common [23-26], and multiple pregnancy rates were also increasing [22,24,27]. The proportion of induced labors was noted to increase over a similar time period to the observed increase in PPH (Figure 3) [13,23,25,28].

As none of the datasets examined had information on potential environmental exposures, we conducted a literature search to investigate these factors. Using Pubmed search terms to investigate possible associations between environmental contaminants, toxins or environmental toxins, alternative/complementary medicine, antidepressants and postpartum hemorrhage failed to identify environmental factors that may have been responsible for recent increases in postpartum hemorrhage.

## Discussion

### Definitions of PPH

While no single definition for PPH is promulgated for clinicians, in the United States and Canada, for example, a blood loss of 500 mL for a vaginal delivery and 1,000 mL for a caesarean birth are often used [5,29]. In contrast, a blood loss of 500 mL for a vaginal delivery and 750 mL for a caesarean delivery is used in Australia [8], and in other data 500 mL blood loss is used to define PPH irrespective of the mode of delivery. Regardless of the definitions used, routine visual estimates of blood loss are frequently inaccurate [30,31], and analyses using calculated blood loss demonstrate that many and perhaps most women lose enough blood at delivery to meet the diagnostic criteria for PPH [31,32]. Moreover, while women delivered by caesarean section lose more blood on average than women who have vaginal births, there is no reason to believe that the physiologic impact of blood loss differs according to the route of delivery, suggesting that the use of a unified definition irrespective of the route of delivery is more appropriate. Alternatively, PPH has been defined as a 10% or more drop in hematocrit [33]. How these definitions are used, their inherent inaccuracies, and the translation of definitions to administrative ICD coding complicates the interpretation of trend data. For example, instructions to medical coders may not discriminate between modes of delivery and instead use 500 mL of vaginal blood loss regardless of delivery route.

The interpretation of coded data may also be limited by the codes themselves. One important problem with the current ICD codes is that the code O72.1 does not allow a distinction to be made between atonic PPH and other forms of PPH that occur in the first 24 hours following delivery of the placenta (such as that due to genital trauma). Also, a single code is available for all types of retained, trapped and adherent placenta with hemorrhage (O72.0). Separate codes for adherent placenta may be useful given increases in the frequency of caesarean delivery; such a code was added to the ICD10 - Australian modification in 2002 (O43.2* Morbidly adherent placenta including placenta accreta, increta and percreta), enabling subsequent study in that population [34].

The use of blood transfusions and procedures to control bleeding have been used as markers of the severity of PPH and to identify women with severe pregnancy morbidity [5,15,16,35,36]. In Australia, Scotland and the USA, increases in the reported rates of severe complications of childbirth have been almost entirely due to reported increases in the use of blood transfusions and/or severe obstetric hemorrhage [15,16,35,36]. In these countries it appears that not only are PPH rates increasing but so is the hemorrhage severity. In contrast, Canadian rates of severe maternal morbidity remained stable between 1991 and 2000, in the context of comparatively low and stable rates of transfusion [5,37]. International differences may reflect differing attitudes among obstetricians about blood transfusions.

#### Recommendation 1

Future revisions of the International Classification of Diseases should include separate codes for atonic PPH and PPH immediately following childbirth that is due to other causes. Also, additional codes are required for placenta accreta/percreta/increta.

#### Recommendation 2

Definitions of PPH should be unified; further research is required to investigate how definitions are applied in practice to the coding of data.

### Improvements in data collection

Currently collected data do not allow us to adequately categorize PPH according to severity and therefore to determine outcomes for women with differing degrees of blood loss. Future research to investigate outcomes and relate these to management, and thus to generate recommendations for improved practice will therefore require improvements to current data collection. These will include, as discussed above, improvements to the codes themselves and to the training for coders. By recording actual estimated blood loss, for example using a simple blood collector bag [38], details of the management of the third stage including the dose, timing and route of prophylactic oxytocic administration, as well as operative procedures and therapies undertaken to control PPH, for example, use of additional uterotonic therapy, intramyometrial prostaglandin administration, brace sutures, intrauterine balloon tamponade, uterine vessel ligation or embolization, we can begin to generate the robust evidence required to develop appropriate clinical guidelines.

#### Recommendation 3

Additional improvement in the collection of data concerning PPH is required, specifically including a measure of severity.

### Associated factors

#### Population characteristics

In all populations examined, maternal age at childbirth was increasing [22-25]. Although we were not able to demonstrate an impact on PPH rates [5,6,39], increasing maternal age is known to be a risk factor for hysterectomy for peripartum hemorrhage [5,12,40]. In the UK and Australia, births to immigrant women are increasing [22,23,41]; rates of severe maternal morbidity, although not specifically PPH, have been shown to be higher in women from ethnic minority groups [36,42]. Rising rates of obesity, demonstrated in many countries [43-47], may also impact on the incidence of PPH; raised Body Mass Index (BMI) is a reported risk factor for hemorrhage [48,49].

#### Obstetric practice

Caesarean delivery is becoming more common globally [23-26], and is known to lead to a higher blood loss than normal delivery, as reflected in differences according to mode of delivery in the threshold level of blood loss used to define PPH [4,50]. However, validation of data on PPH from New South Wales showed that there is significant under-recording of blood loss after caesarean delivery (60% of caesarean deliveries with recorded blood loss versus 96% of vaginal births) [51]. Additionally, post-caesarean transfusion for low HB or post-CS laparotomy for evacuation of hematoma are not captured in the PPH code [52], which may explain the lack of an observed increase in risk of PPH in women undergoing CS delivery in this population. Other studies have shown that previous delivery by caesarean section is associated with increased risk of abnormal placentation, hemorrhage and peripartum hysterectomy [5,12,53]. In Canada, where PPH following caesarean delivery requires a blood loss over 1,000 ml, caesarean delivery had a protective effect on atonic postpartum hemorrhage (adjusted odds ratio 0.52 (95% CI 0.51-0.53) [5].

Induction of labor is also now practiced more commonly (Figure 3) [13,23,25,28]. Induced labor in standard primiparae in Victoria was associated with increased odds of PPH (OR 1.17, 95%CI 1.04-1.3) [54] (the standard primipara is a 20 to 34 year-old woman, giving birth for the first time, who is free of obstetric and specific medical complications and pregnant with a singleton term pregnancy with a non small for gestational age infant and a cephalic presentation). In addition, after adjustment for mode of delivery, maternal age, birthweight and public/private admission status, use of syntocinon infusion for augmentation also independently increased the odds of PPH (aOR 1.19, 95% CI 1.1-1.3) [54]. A population-based Norwegian study also reported an increase in the risk of severe postpartum hemorrhage associated with induction of labor (aOR 1.71, 95% CI 1.56-1.88) [55]. However, further analysis of data on peripartum hysterectomy to control PPH in the UK did not show an association with induction of labor even after adjustment for previous caesarean delivery (adjusted odds ratio (OR) 1.09, 95%CI 0.70-1.71). Labor induction and other changes in obstetric practice may lead to an increased duration of labor, in both first and second stage, which may contribute to an increase in the frequency of PPH. An increasing duration of labor over time has been shown in Victoria, Australia and Nova Scotia, Canada [56]. The Nova Scotia study found an increase in the risk of postpartum hemorrhage with increasing duration of the second stage of labor. A role for increased labor duration in PPH is also supported by evidence from the UK study of hemorrhage-associated peripartum hysterectomy, which showed an independent association between peripartum hysterectomy and labor of 12 hours or greater duration, adjusted for the effects of age, parity, previous CS delivery, other uterine surgery and multiple pregnancy (adjusted OR 3.04, 95%CI 1.52-6.08). This is also supported by data on atonic PPH in Canada, which shows an increased risk with prolonged first stage (OR 1.49, 95% CI 1.44 -1.55), prolonged second stage (OR 2.13, 95% CI 2.09-2.17) and prolonged labor (unspecified) (OR 1.21, 95% CI 1.11-1.33) [5].

Multiple pregnancy rates are also increasing [22,24,27]; possible contributory factors include assisted reproductive techniques and an aging population of women giving birth. Multiple pregnancy has been shown to be associated with an increased risk of PPH and associated complications in a number of studies [5,12,57,58] and thus the observed rise in the rate of multiple pregnancy may contribute to increasing PPH incidence. However, although there was a significant increase in multiple pregnancies in NSW between 1994 and 2002 (1.4% to 1.7% of all pregnancies, p < 0.001), there was no significant change in the proportion of postpartum hemorrhages among multiple pregnancies. The postpartum hemorrhage rate among multiple pregnancies varied from 2.5% in 1994 to 3.1% in 1996 and 2002 (with an average rate of 2.9%), representing an increase of 183 pregnancies, which in the overall context of postpartum hemorrhage risk was thought to be inconsequential [6].

Analysis of Canadian PPH data showed that adjustment for caesarean delivery and labor induction, several other maternal characteristics and obstetric practices did not explain the increase in PPH rates though it did explain some of the increase in hysterectomy for PPH [5].

#### Accounting for changes in risk factors

Attempts to explain the increase in PPH rates by taking into account changes in the observed risk factors for PPH over time cannot explain the rise in rates [5,6]. Ford et al investigated risk factors for any PPH among singleton deliveries in Australia over the period 1994-2002 [6], while Joseph et al investigated risk factors for atonic PPH among deliveries in Canada over the period 1991-2004 [5]. Using different methods the two studies took into account maternal age, parity, year of birth, country of birth, onset of labor, mode of delivery, epidural analgesia, abnormal labor (precipitate labor, incoordinate contractions), prolonged or obstructed labor, hypertensive disorders, placental abnormalities (placenta or vasa previa), placental abruption, gestational age, birthweight, perineal trauma, cervical laceration, previous caesarean, multiple pregnancy, polyhydramnios, and amniotic cavity infection. Both studies concluded that although the frequency of many risk and protective factors for PPH changed during the study periods, controlling for these factors did not alter temporal trends, suggesting factors other than those considered were responsible for the rising PPH rates.

The authors postulated that other factors such as a more liberal approach to duration of labor which allows women to labor for longer (information unable to be collected with sufficient detail), increases in obesity (not recorded in hospital data) or changes in the management of third stage of labor (not recorded in hospital data) may play a part in rising PPH rates. Other possible risk factors for further investigation could include the effect of induction of labor taking into account agents used or more complex interactions of risk factors such as the interplay between body mass index, oxytocic agents and blood loss or BMI, third stage management and blood loss.

Better and comparable data, particularly where ascertainment is available from multiple datasets, will help resolve some of the limitations of studies relying on hospital discharge data which may under-ascertain risk factors such as obstructed labor [59], lack information on other possible risk factors such as body mass index and sociodemographic factors [60], do not record the subtleties of timing and severity [61] and may not accurately capture antenatal history [62].

#### Recommendation 4

Further research is required to determine whether an increased rate of reported PPH is also observed in other countries, and to further investigate potential risk factors including increased duration of labor, obesity and changes in second and third stage management practice.

### Diagnosis and management of PPH

Many of the trials and interventions in the management of postpartum hemorrhage have concentrated on the implementation of drills and treatments [63]. However, the diagnosis of hemorrhage is of primary concern and the importance of the accurate estimation of blood loss is paramount to allowing the appropriate intervention by warning of impending hemorrhagic shock. Estimates of blood loss by paramedics [64], surgeons [65] and obstetricians and midwives [66] are often inaccurate and vary widely. Studies following vaginal [67] and abdominal delivery [68] show visual estimation of blood loss to be of limited clinical use.

Bose et al [66], demonstrated a marked discrepancy between estimated and actual blood loss at varying volumes confirming the clinical difficulty in accurately estimating blood loss, particularly in obstetric scenarios. They developed a simple algorithm to facilitate visual estimation and felt that these along with clinical reconstructions provided a popular and useful learning tool to facilitate the visual estimation of blood loss. This has been confirmed [69].

In the post partum period, particularly after caesarean section, the blood loss may not be visible and accurate estimation of the hemodynamic state is important. The adoption of Early Warning Systems [70] to obstetric practice (Modified Obstetric Early Warning Scoring System - MOEWS), which allows easy assessment of trends in hemodynamic assessments and impending shock, is now widespread. These take various forms but utilize colored blocks to signify worrying trends in pulse, blood pressure or temperature measurements. This should lead to earlier intervention but full evaluation is awaited.

#### Recommendation 5

Training should be provided to all staff involved in maternity care concerning assessment of blood loss and the monitoring of women after childbirth using, for example, the Modified Obstetric Early Warning Scoring (MOEWS) system. This is key to reducing the severity of PPH and preventing any adverse outcomes.

#### Recommendation 6

Clinicians should be more vigilant given the possibility that the frequency and severity of postpartum hemorrhage has in fact increased. This applies particularly to small hospitals with relatively few deliveries where management protocols may not be defined adequately and drugs or equipment may not be on hand to deal with unexpected severe PPH.

### Strengths and limitations of this review

An important strength of this study is that we have reviewed the available data on PPH across a range of high resource countries and considered potential associated factors where there are data available. However, we did not review data from low income countries, and thus the conclusions we draw may not necessarily be applicable to these settings. In order to include as much information as possible, we included data in which varying definitions of PPH were used, as discussed above, which may also be regarded as a limitation. We believe, however, that the inclusion of a wide range of data sources has allowed us to generate comprehensive recommendations for further study and management of this condition.

Since, for the majority of countries, comprehensive individual level data were not available, we have not been able to investigate whether there have been temporal changes in the proportion of women experiencing PPH with or without accompanying risk factors. Nevertheless, we have demonstrated that changes in numerous known risk factors over time have not explained increases in postpartum hemorrhage in at least 2 settings and that there are a number of associated changes in risk factors which may warrant investigation in future ecological or other correlation studies.

## Conclusion

### Key Recommendations

1. Future revisions of the International Classification of Diseases should include separate codes for atonic PPH and PPH immediately following childbirth that is due to other causes. Also, additional codes are required for placenta accreta/percreta/increta.

2. Definitions of PPH should be unified; further research is required to investigate how definitions are applied in practice to the coding of data.

3. Additional improvement in the collection of data concerning PPH is required, specifically including a measure of severity.

4. Further research is required to determine whether an increased rate of reported PPH is also observed in other countries, and to further investigate potential risk factors including increased duration of labor, obesity and changes in second and third stage management practice.

5. Training should be provided to all staff involved in maternity care concerning assessment of blood loss and the monitoring of women after childbirth using, for example, the Modified Obstetric Early Warning Scoring (MOEWS) system. This is key to reducing the severity of PPH and preventing any adverse outcomes.

6. Clinicians should be more vigilant given the possibility that the frequency and severity of postpartum hemorrhage has in fact increased. This applies particularly to small hospitals with relatively few deliveries where management protocols may not be defined adequately and drugs or equipment may not be on hand to deal with unexpected severe PPH.

## Competing interests

The authors declare that they have no competing interests.

## Authors' contributions

MK drafted the manuscript/paper; CB and WC helped obtain funding, organize and facilitate the workshop; all authors actively participated in the workshop, presented data, contributed to discussions and participated in revising the manuscript and reviewing the final version for submission.

## Pre-publication history

The pre-publication history for this paper can be accessed here:

http://www.biomedcentral.com/1471-2393/9/55/prepub
